# Daridorexant treatment for chronic insomnia: a real-world retrospective single-center study

**DOI:** 10.1007/s10072-024-07326-w

**Published:** 2024-01-27

**Authors:** Mariana Fernandes, Fabio Placidi, Nicola Biagio Mercuri, Claudio Liguori

**Affiliations:** 1 Department of Systems Medicine, University of Rome Tor Vergata, Rome, Italy; 2 Sleep Medicine Centre, Neurology Unit, University Hospital of Rome Tor Vergata, Rome, Italy

**Keywords:** Chronic insomnia disorder, Daridorexant, Dual orexin receptor antagonist, Insomnia Severity Index

## Abstract

**Introduction:**

Chronic insomnia disorder (CID) significantly impacts well-being and daily functioning. Daridorexant, a double orexin receptor blocker, has shown efficacy in randomized clinical trials and has been recently approved for the treatment of CID in adult patients. This retrospective observational study aimed to describe real-world data on daridorexant effectiveness and safety in adult patients with CID.

**Methods:**

Consecutive patients initiating on-label daridorexant at the Sleep Medicine Centre, University Hospital of Rome Tor Vergata were enrolled. Baseline and 30-day follow-up (FU) evaluations included patients’ and CID characteristics, comorbidities, and clinicians’ and patients’ subjective ratings of changes with the Clinical and Patient Global Impression-Improvement scores (CGI-Is and PGI-Is), as well as Insomnia Severity Index (ISI) scores in a subgroup of patients.

**Results:**

Sixty-nine patients initiated 50-mg daily dosage. At FU, 58% of both patients and clinicians rated CID as improved on CGI-Is and PGI-Is, with no differences based on comorbidities, sex, or number of previous medications. No significant predictors of CGI-Is and PGI-Is improvement were identified. At FU, ISI scores (*n* = 24) significantly decreased from 18.25 ± 3.21 to 12.08 ± 6.12 (*Z* = 8.000; *p* < 0.001). Of these, eight patients (33.3%) had absence of insomnia symptoms, and no patients reported a worsening in ISI score categories.

**Conclusions:**

This study suggests daridorexant to be effective and safe in real-world CID treatment whether used as a first-ever treatment, switch, or add-on, as reflected by subjective and objective measures and the absence of serious treatment-related adverse events. Future research on larger cohorts should explore daridorexant potential across diverse patient characteristics.

## Introduction

Insomnia is a common condition within the general population, being the second most prevalent mental disorder and the most common sleep complaint. Chronic insomnia disorder (CID) is a prevalent sleep disorder significantly affecting individuals’ well-being and daily functioning, affecting about 10–20% of the population, especially women, older adults, and individuals of lower socioeconomic status [1]. Insomnia can be very heterogeneous in terms of clinical presentation, patients’ complaints, and underlying mechanisms and is often associated with comorbid conditions; moreover, it can precipitate the patients’ quality of life (QoL) and overall health and is a risk factor for other neurological conditions such as epilepsy, headache, and Alzheimer’s and Parkinson’s diseases [1﻿].

It is thus crucial to treat insomnia to improve patients’ QoL and general health. One of the most recent therapeutic options takes advantage of the orexin system by blocking orexin receptors. This is of particular interest as orexins act on different biological circuits, both centrally and peripherally, involved in insomnia and other associated conditions [2]. Among these new drugs, daridorexant (a dual orexin receptor antagonists) has been recently approved for the treatment of adult patients with insomnia in North America and Europe [3–8]. Several randomized clinical trials (RCTs) showed that daridorexant significantly improved subjective and objective sleep measures, as well as daytime functioning in the short- (up to 3 months) [9–13] and long-term (up to 1 year) [14]. Daridorexant has good efficacy and tolerability, has limited potential for abuse and rebound effects after discontinuation, and does not negatively impact cardiac and respiratory parameters during nighttime sleep; these characteristics make it particularly useful even in cases of polypharmacy—such in the elderly—and substance misuse [2].

This study aimed to evaluate retrospective data on the effectiveness and safety of daridorexant treatment in adult patients with CID, as to provide evidence from real-world clinical practice.

## Methods

The present investigation is a retrospective observational study. Consecutive patients starting on-label daridorexant 50 mg/daily per clinician’s choice at the Sleep Medicine Centre, Neurology Unit, University Hospital of Rome Tor Vergata were enrolled and evaluated at baseline and at 30-day follow-up (FU). We evaluated patients’ characteristics, CID duration, previous insomnia treatments, comorbidities, and clinicians’ and patients’ subjective ratings of changes with the Clinical and Patient Global Impression-Improvement scores (CGI-Is and PGI-Is) that are rated on 7-point Likert scale (1 = very much improved; 7 = very much worse). Changes in insomnia symptoms were evaluated with the Insomnia Severity Index (ISI) scale in a subgroup of patients.

### Statistical analysis

Data analysis was. Categorical data are reported as counts and percentages. The significance group differences conducted with the statistical program SPSS for Windows version 25.0 (IBM Corp, Armonk, NY, USA). Data are reported as mean ± standard deviation in CGI-I and PCG-I outcomes were evaluated with the Mann–Whitney *U* test and Kruskal–Wallis test. The association of CID duration with CGI-I and PGI-I was explored through Kendall’s tau correlation analysis. Two sets of hierarchical regression analyses accounting for the respective contribution of age, CID duration, and number of previous insomnia medications were performed considering CGI-I and PGI-I as outcomes. *p*-values < 0.05 indicate statistical significance.

## Results

Sixty-nine patients were enrolled (see Table 1 for patients’ characteristics), all starting treatment with a 50-mg daily dosage. CGI-Is and PGI-Is are available for the entire cohort. At FU, mean PGI-Is were 3.00 ± 1.25, namely, only 5 (7.2%) patients rated their CID as worsened, while it remained unchanged for 24 (34.8%) and improved for 40 (58.0%). Mean CGI-Is at FU were 2.80 ± 1.18, namely, clinicians never rated insomnia as worsened, while they rated it as unchanged for 29 (42.0%) and improved for 40 patients (58.0%). No differences in CGI-Is (*χ*^2^ = 1.591, *p* = 0.451) and PGI-Is (*χ*^2^ = 1.079, *p* = 0.583) according to the number of comorbidities and most common comorbidities were found (depression (*U* = 585.0, *p* = 0.266; *U* = 623.5, *p* = 0.107, respectively); anxiety (*U* = 447.50, *p* = 0.936; *U* = 443.0, *p* = 0.989, respectively)). Moreover, no differences in CGI-Is and PGI-Is across sexes (*U* = 562.50, *p* = 0.690; *U* = 579.50, *p* = 0.857, respectively), number of previous medications (*χ*^2^ = 5.064, *p* = 0.281; *χ*^2^ = 5.117, *p* = 0.276, respectively), and previous insomnia treatment medication use (*p* = 0.982; *p* = 0.610) were found. Further, no association between CID duration and CGI-I (τb =  − 0.012, *p* = 0.900) and PGI-I (τb =  − 0.047, *p* = 0.613) was found. In linear regression models, age (*β* = 0.030, *p* = 0.817; *β* = 0.010, *p* = 0.352, respectively), CID duration (*β* = 0.011, *p* = 0.930; *β* =  − 0.076, *p* = 0.547, respectively), and number of previous insomnia treatment medication use (*β* =  − 0.009, *p* = 0.946; *β* = 0.134, *p* = 0.303, respectively) did not predict CGI-I (*F*_3,68_ = 0.027, *p* = 0.994) and PGI-I (*F*_3,68_ = 0.561, *p* = 0.643). The model explained 3.5% of the variance for CGI-I and 15.9% of the variance for PGI-I. At FU, 12 (17.4%) dropped out, of which five (7.3%) due to inefficacy, and seven (10.2%) due to personal reasons.

**Table 1:** Patients’ characteristics and comorbidities, chronic insomnia disorder (CID) type and duration, and previous insomnia medication use

Patients enrolled	69
Sex, *n* (%)	
Male	36 (52.2)
Female	33 (47.8)
Age (years)	
Mean ± sd	51.32 ± 16.42
Range	19–83
Type of insomnia, *n* (%)	
Sleep maintenance insomnia	50 (72.5)
Sleep onset insomnia	11 (15.9)
Both	8 (11.6)
CID duration (months)	
Mean ± sd	59.97 ± 69.45
range	3–360
Number of previous insomnia medications, *n* (%)	
0	9 (13.0)
1	15 (21.7)
2	19 (27.5)
3	7 (10.1)
4 or more	19 (27.5)
At least one	60 (87)
Patients not on treatment and starting daridorexant, *n* (%)	9 (13)
Patients with direct switch to daridorexant, *n* (%)	34 (49.3)
Patients using daridorexant as an add-on, *n* (%)	26 (37.7)
Number of comorbidities, *n* (%)	
0	19 (27.5)
At least 1	34 (49.3)
At least 2	16 (23.2)
Most common comorbidities, *n* (%)	
Depression	21 (30.4)
Anxiety	17 (24.6)
Epilepsy	6 (8.7)
Mild cognitive impairment or dementia	5 (7.2)

ISI evaluations were available for 24 patients. At FU, ISI scores significantly decreased from 18.25 ± 3.21 to 12.08 ± 6.12 (*Z* = 8.000; *p* < 0.001). Moreover, eight patients (33.3%) had absence of insomnia symptoms (including two with severe baseline ISI scores), seven (29.17%) reported mild symptoms, and nine (37.5%) referred symptoms of moderate severity (including one with severe baseline ISI score). Of the 18 patients with moderate ISI scores at baseline, ten (55.56%) showed improvements (four (22.22%) had no clinically significant insomnia; six (33.33%) had mild symptoms), whereas the remaining eight (44.44%) remained unchanged. Considering the three patients with mild severity ISI scores at baseline, two (66.67%) showed no clinically significant insomnia, and one (33.33%) remained in the mild category. Finally, no patients reported a worsening in ISI scores categories at FU.

## Discussion

This study retrospectively evaluated the real-world daridorexant effectiveness and tolerability in unselected patients suffering from CID, mainly sleep maintenance insomnia. All patients received a 50-mg daily dosage, as this was shown to be better in improving insomnia symptoms and daytime functioning compared to lower doses [9, 10, 13]. In the subset of patients with available ISI, scores decreased significantly in 30 days. This objective change was accompanied by favorable physicians’ and patients’ perceptions of improvement as measured by CGI-Is and PGI-Is. Namely, almost 60% of both patients and clinicians judged CID as improved, while the rest rated it as unchanged, except for a small subset of five patients who rated it as worsened. Benefits were observed irrespective of whether daridorexant was used as the first-ever insomnia medication, as a switch, or as an add-on; Table 2 provides three illustrative cases of daridorexant use in these settings. No patient dropped out of the study due to treatment-related adverse events (TAE), indicating daridorexant safety.

**Table 2:** Three illustrative cases of daridorexant used as first-ever insomnia treatment, direct switch from other drugs, or as an add-on. The switching approach used was as follows: case 2, direct switching featured by starting daridorexant and concomitantly stopping (melatonin) or tapering down for discontinuation the previous treatment (i.e., sedative antidepressant—trazodone, Z-drugs, benzodiazepines) and case 3, add-on treatment featured by adding on daridorexant to previous treatments for insomnia and deciding to tapering down and discontinuing the previous treatments (sedative antidepressant—trazodone, Z-drugs, benzodiazepines) only at the follow-up visit. All patients received psychoeducation about sleep hygiene during the visit on top of pharmacological treatment

	Daridorexant-naïve patient (case 1)	Direct switch to daridorexant (case 2)	Daridorexant as an add-on (case 3)
Patient’s sex and age	• 50-year-old male	• 50-year-old female	• 68-year-old female
Insomnia background	• Maintenance insomnia • **Baseline ISI: 13** • Does not acknowledge sleep disturbance	• Maintenance insomnia symptoms for 2 years • **Baseline ISI: 21** • Unsuccessful benzodiazepine treatment	• Mixed insomnia (both initial and maintenance) persisting for > 30 years • **Baseline ISI: 21** • No nocturnal apneas and RLS history • Previous treatments with triazolam, lormetazepam, melatonin, and trazodone without benefits
Medical history	• Concussive head trauma due to a road accident during sleep deprivation and a state of stress • No abnormalities in MRI, EEG, and objective neurological assessment	• Hashimoto’s disease (no treatment required) • Irregular menstrual cycles • Mild depression symptoms associated with anxiety and tendency to be hypervigilant	• Psoriatic arthropathy • Fibromyalgia • Hypothyroidism • Sinus tachycardia • Dyslipidemia • Glaucoma
Sleep schedule	• Nocturnal awakenings • Reduction in the total time spent sleeping (4–5 h/night; 7/7 nights; time in bed, 8–8.5 h; sleep efficiency, ~ 50%)	• Bedtime: ~ 22:30 • Sleep onset: < 30 min • Sleep maintenance: wakes around 3–4 h after sleep onset • Stays in bed trying to fall asleep without effects	• Bedtime: ~ 23:30 after taking zolpidem 5 mg • First awakening after ~ 2 h, then takes zolpidem 5 mg and sleeps for another ~ 3 h. Wakes up at 7:30, with no refreshing sleep
Daytime symptoms	• Agitated and tense during the day • Afraid to become addicted to insomnia medications	• Fatigue (especially post-lunch) • Migraine (< 1 episode a month)	• Non-restorative sleep • Daytime sleepiness • Fatigue
Treatment at baseline	• None	• Trazodone 25 mg	• Zolpidem 5 mg taken twice at night; trazodone 75 mg; pregabalin 75 + 150
Treatment at FU	• Daridorexant 50 mg/day	• Daridorexant 50 mg/day • Trazodone tapered down by 25% weekly reduction using drops and completely discontinued in 4 weeks	• Same as above, but o Discontinued the dose of zolpidem during the night following psychoeducation about sleep o Added daridorexant 50 mg/day
FU assessment	• **ISI score**: 3 (10-point improvement) • GCI-I: 1 • PGI-I: 1 • The patient reports improvement in night sleep, going to bed at 23, falling asleep within 20 min, and getting up at 7	• **ISI score:** 7 (14-point improvement) • Now reports sleeping for ≥ 6 consecutive hours • Some awakenings in the night (2 per week), but can fall asleep again immediately • Patient’s treatment preference: maintaining daridorexant	• **ISI score**: 12 (9-point improvement) • GCI-I: 1 • PGI-I: 1 • At 30-day FU visit trazodone is reduced for stopping by 25 mg every 2 weeks and completely discontinued in 5 weeks • Zolpidem 5 mg before going bed is maintained (hypothetical reduction or stop at the newly planned FU visit in the following 2 months)

*CGI-I* Clinical Global Impression-Improvement scores, *EEG* electroencephalogram, *FU* follow-up, *ISI* Insomnia Severity Index, *MRI* magnetic resonance imaging, *PGI-I* Patient Global Impression-Improvement scores, *RLS* restless leg syndrome

Our results concord with the literature. Daridorexant has been evaluated in five RCTs (and post-hoc analyses), which showed its efficacy in improving several sleep outcomes such as reducing objective wake after sleep onset (WASO) and sleep latency (SL) measured after 1–2-day and 1–3-month FU, as well as improving subjective total sleep time (sTST) at 1 month [10]. Moreover, TAEs were never serious and were comparable to the placebo groups [9–14]. One long-term RCT showed daridorexant treatment for up to 12 months to be safe and well tolerated, resulting in sustained improvements in sleep and daytime functioning without concerns of late-emerging AEs [14].

Real-world studies also highlight daridorexant favorable profile in several objective scales, namely by significantly improving ISI scores (mean reduction of 7.0 ± 0.54 points) and subjective sleep parameters, such as sTST (mean increase of 54 ± 1.0 min), sSL (mean decrease of 23.9 ± 2.4 min), sWASO (mean decrease of 31.6 ± 3.2 min), and sleep efficiency (mean improvements of 10.5 ± 1.1%) [15]. Preliminary data from two prospective observational studies show that 1–3 months of daridorexant treatment result in improvements in health-related QoL [16], as well as in mood, anxiety, suicidal risk measures, and in the ability to regulate emotions and to reduce dysfunctional sleep beliefs which, in turn, may fuel hyperarousal [17].  Our cohort was also characterized by the presence of several comorbidities—especially depression, anxiety, epilepsy, and cognitive impairment—and polypharmacy. The results of the present study coupled with the other recent observations [17]  are of particular interest as the enrolled patients with comorbid mood or anxiety disorder (or both) were taking antidepressants and/or mood stabilizers, and these findings may support the use of daridorexant in treatment-resistant CID in concomitance with polypharmacological treatments. The lack of differences in CGI-Is and PGI-Is across sexes, number of previous medications and comorbidities, and CID duration in our study, as well as the improvements in mood and emotions scores in the study by Palagini et al., suggest that daridorexant is effective regardless of the setting. For instance, in this study, daridorexant treatment was prescribed also in elderly patients with mild cognitive impairment, a group often characterized by sleep impairment [18], frailty, and multiple drug intake, substantiating the previous evidence suggesting a possible role of orexin neurotransmission downregulation for improving sleep in patients with cognitive impairment [19, 20] and showing the safety and efficacy of the drug with no increased risk of TAEs or residual effects the next morning, especially with the 50-mg dosage [12, 13]. Moreover, patients with comorbid epilepsy and insomnia were also evaluated in this study, and considering the proposed rational for the orexin receptors block in epilepsy [21], the beneficial effects of daridorexant may suggest new therapeutic targets for improving sleep and epilepsy in those patients [22].

Daridorexant efficacy and safety are probably due to its pharmaco-dynamic and pharmaco-kinetic properties. As a dual orexin receptor antagonist, daridorexant improves insomnia by selectively binding to both orexin receptors rather than inducing global sedation like GABA receptor agonists [8]. Furthermore, it presents no bioaccumulation and active metabolites, it is absorbed quickly, and its half-life is optimal for an insomnia medication (∼ 8 h) [8, 23, 24]. Its safety profile is characterized by the absence of serious both short- and long-term TAEs. Moreover, it is accompanied by the lack of withdrawal symptoms or rebound insomnia [9, 10, 14], in contrast to benzodiazepine and Z-drugs, which show dependency and drug-liking concerns [8].

### Limitations

The present study has several limitations, especially those related to a retrospective observational design such as selection bias, limited control and accounting for confounding variables, and the inability to establish causal relations. Moreover, the relatively small number of enrolled patients might have hindered subgroup statistical comparisons and linear regressions. Finally, ISI scores were evaluated only in a small subset of patients.

Further studies on larger cohorts, both observational and RCTs, are warranted to explore the potential and limitations of daridorexant treatment according to patients’ and CID’s characteristics, comorbidities (especially psychiatric and neurological), and concomitant drug use. Polysomnographic studies addressing sleep architecture changes with daridorexant treatment are scarce and warranted and will be of use in increasing our understanding of how daridorexant actually exerts its clinical benefits. To date, it seems that dual orexin receptor antagonists improve sleep by increasing REM sleep and could thus be of particular interest in those conditions where REM sleep is particularly disrupted such as for anxiety and stress-related disorder [25].

## Conclusion

Clinical and patients’ ratings of overall improvement at FU (CGI-I and PGI-I), significant reductions in the ISI scores, and lack of TAEs indicate that daridorexant is effective and safe in treating adult patients with CID in a real-world setting. Patients’ and insomnia’s features seem to not have affected treatment in our sample. Larger cohorts are warranted to further explore daridorexant potential and differences in clinical practice according to patients’ features.

## Data Availability

Raw data can be provided by the corresponding author upon reasonable request.
